# A Tendon-Specific Double Reporter Transgenic Mouse Enables Tracking Cell Lineage and Functions Alteration In Vitro and In Vivo

**DOI:** 10.3390/ijms222011189

**Published:** 2021-10-17

**Authors:** Rui Chen, Xunlei Zhou, Thomas Skutella

**Affiliations:** Department of Neuroanatomy, Group for Regeneration and Reprogramming, Institute for Anatomy and Cell Biology, Medical Faculty, Heidelberg University, 69120 Heidelberg, Germany; rui.chen@uni-heidelberg.de

**Keywords:** tendon tissue engineering, repair, animal model, decellularized extracellular matrix, mechanical stimulation

## Abstract

We generated and characterized a transgenic mouse line with the tendon-specific expression of a double fluorescent reporter system, which will fulfill an unmet need for animal models to support real-time monitoring cell behaviors during tendon development, growth, and repair in vitro and in vivo. The mScarlet red fluorescent protein is driven by the *Scleraxis* (*Scx*) promoter to report the cell lineage alteration. The blue fluorescent protein reporter is expressed under the control of the 3.6kb *Collagen Type I Alpha 1 Chain* (*Col1a1*) proximal promoter. In this promoter, the existence of two promoter regions named tendon-specific cis-acting elements (TSE1, TSE2) ensure the specific expression of blue fluorescent protein (BFP) in tendon tissue. Collagen I is a crucial marker for tendon regeneration that is a major component of healthy tendons. Thus, the alteration of function during tendon repair can be estimated by BFP expression. After mechanical stimulation, the expression of mScarlet and BFP increased in adipose-derived mesenchymal stem cells (ADMSCs) from our transgenic mouse line, and there was a rising trend on tendon key markers. These results suggest that our tendon-specific double reporter system is a novel model used to study cell re-differentiation and extracellular matrix alteration in vitro and in vivo.

## 1. Introduction

Tendon, especially the Achilles tendon, is one of the musculoskeletal systems most vulnerable tissues to ageing and injury [1]. The development of the tendon usually involves several key stages, including the embryonic, newborn and the adult stage [2]. In turn, tendon repair usually consists of three stages: the inflammatory, proliferative, and the remodeling stages [3]. Transitions between cell lineages and changes in the extracellular matrix occur at various of these stages throughout tendon development, ageing, and post-injury repair. Therefore, continuous observation of cell identity and production of extracellular matrix is crucial to the study of the tendon healing process.

The basic helix-loop-helix (b-HLH) transcription factor *Scleraxis* (*Scx*) is a specific marker for tendon cells [4]. In studies in which *Scx* directs lacZ expression, it was observed that early embryonic (pre-E13.5) *Scleraxis* expression is widespread in several organs, including bone, heart, and lung [5]. However, after E13.5, *Scx* expression becomes specific and is mainly expressed in tendons and ligaments [4,6]. Moreover, *Scleraxis* expression increases after injury [7]. Therefore, constructing a *Scleraxis* reporter mouse line could provide an ideal model for cell identity changes during tendon development, ageing, and healing. However, *Scx* fluorescent reporter mouse lines are currently available (e.g., *Scx*-GFP); the limitations of this transgenic tendon reporter mouse are the use of partial regulatory elements of the *scleraxis* gene (extends ∼4 kb upstream and ∼5 kb downstream of the *Scx* gene) [8]. This design of transgenic constructs missing two presumed repressor elements leads to the expression of GFP (green fluorescent protein) being broader than the endogenous *Scx* expression [8]. As such, we built a transgenic construct with an approximately 14 kb genomic clone (which includes an about 5 kb upstream promoter and an about 6 kb downstream enhancer).

Type I collagen is the principal tendon component that provides mechanical strength to the tendon [9]. This means collagen I is an ideal marker for monitoring changes in the tendon extracellular matrix. However, the widespread expression pattern of *Col1a1*(collagen I encoded by the Cola1 gene) limits its application as a tendon-specific marker. The LacZ expression pattern driven by a 3.2 kb promoter of the mouse pro-alpha 1(I) collagen suggests that a cis-acting regulatory DNA element located between 2300 and 3200 bp upstream of the start site directs the tendon and fascia specific expression [10]. Further studies revealed two tendon-specific cis-acting elements (TSE1, TSE2) in this region. The combination of these cis-acting elements (including osteoblasts specific elements) direct LacZ expression specific in tendon [11]. Thus, we generated a tendon-specific transgenic mouse with BFP fluorescence driven by this proximal promoter. By insulators derived from the chicken β-globin locus near the 5′ boundary of the chicken β-globin structural domain, we separated the *Col1a1*-BFP reporter from the genomic DNA to prevent the position effects caused by the insertion site of the transgene in the genome (e.g., aberrant activation) [12].

**Figure 1:**
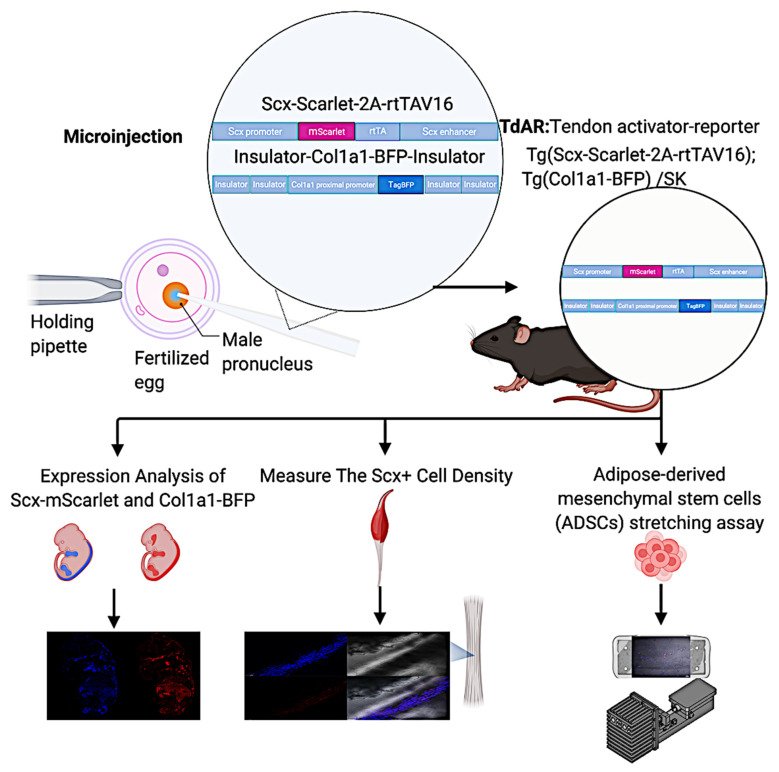
Scheme of this study. Tg (*Scx*-Scarlet-2A-rtTAV16) Tg (*Col1a1*-BFP) /SK mouse line was obtained from pro-nuclear microinjection. Then we analyzed the *Scx*-mScarlet and *Col1a1*-BFP expression in the whole-mounted embryo and cryosection. The Scx+ cell densities in the central region of the Achilles tendon from young and adult mice were measured using Fiji. Further, the adipose-derived mesenchymal stem cells (ADMSCs) stretching assay was performed in vitro. (This figure was created using BioRender.com).

In this study, our goals included (1) to analyze the expression of *Scx*-mScarlet and *Col1a1*-BFP in the embryo; (2) to measure the Scx+ cell density in the central region of the Achilles tendon from young and adult mice; and (3) to validate the tendon-specific double reporter system by adipose-derived mesenchymal stem cells (ADMSCs) stretching assay in vitro (Figure 1).

## 2. Results

### 2.1. Generation of Tendon-Specific Double Transgenic Reporter Mice

The linearized and purified transgenic constructs (*Scx*-mScarlet-2A-rtTAV16 and *Col1a1*-BFP) were mixed at equimolar ratios and microinjected. The microinjection was performed in 297 pro-nuclear fertilized eggs, and 76 mice were obtained (36 females and 40 males). In total, five tendons activator-reporter transgenic mouse (TdAR) lines showed mScarlet and mTagBFP (scientific name mTagBFP is a monomeric blue fluorescent protein) co-expression in the Achilles tendon. We compared the transgenes expression levels of different founders. The expression patterns were analogous but in differential intensity. Therefore, we focused on the offspring of one founder for further characterization, which showed robust reporter expression and no phenotypic consequences.

### 2.2. Expression Profile of Scx-mScarlet and Col1a1-BFP Transgenic Mice

#### 2.2.1. Expression of *Scx*-mScarlet Reporter Is Tendon-Specific

To validate our transgenic mouse model, the mScarlet expression profile verification was carried out. Embryos at different developmental stages were collected and observed under the binocular fluorescence microscope. The expression distribution of red fluorescence was colocalized with tendon tissue at most stages.

**Figure 2:**
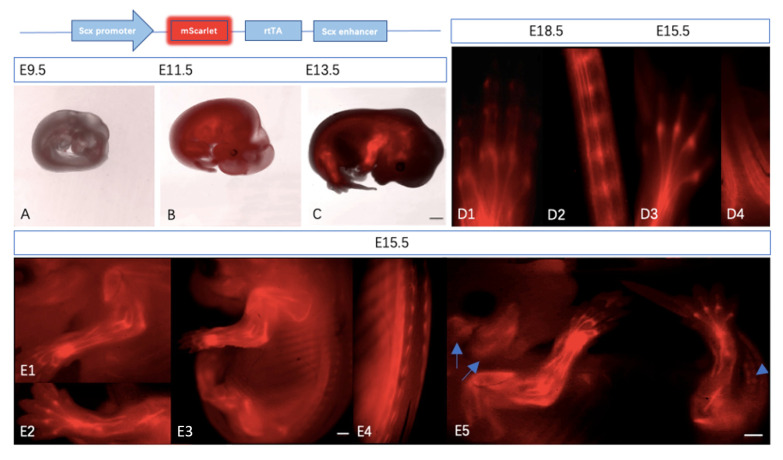
Areas of *Scx*-mScarlet expression coincided with tendon atlas. To characterize *Scx*-mScarlet transgenic mice, we detected the red signal of the whole-mount embryos using the binocular fluorescence microscope. The schematic illustration above indicates the *Scx*-mScarlet transgene construct. (**A**–**C**): *Scx*-mScarlet embryo at E9.5, E11.5, and E13.5, respectively. (**D1**): *Scx*-mScarlet in a skinned hind paw of E18.5 embryo. (**D2**): *Scx*-mScarlet in a skinned tail of E18.5 embryo. (**D3**): *Scx*-mScarlet in the hind paw of E15.5 embryo. (**D4**): *Scx*-mScarlet in the tail of E15.5 embryo. (**E1**): *Scx*-mScarlet in a forelimb of E15.5 embryo. (**E2**): *Scx*-mScarlet in a hindlimb of E15.5 embryo. (**E3**): Lateral views of the *Scx*-mScarlet embryo at E15.5. (**E4**): *Scx*-mScarlet in the ribs of E15.5 embryo. (**E5**): Live whole-mount images of an *Scx*-mScarlet embryo at E15.5 (Scale bar = 500 μm in (**C**,**E3**,**E5**); arrows show tendons around the developing ipsilateral temporalis and masseter muscles; arrowhead indicates the mScarlet-expression in the annulus fibrosus).

The expression of endogenous *Scx* was gradually enhanced starting from E9.5 [13]. At embryonic day (E) 9.5, *Scx* is mainly detected in the branchial arches [8]. *Scx*-positive areas became more restricted to the limited areas in both limb buds and syndectome (a somitic compartment of tendon progenitors) at E11.5 [4]. The Scx+ tendon precursor cells in the limb buds at E11.5 were gradually concentrated to the tendon primordia in limbs, ribs, and head at E13.5 [14]. The mScarlet faithfully recapitulates the above endogenous *Scx* expression profile during tendon development. The reporter is first detected in the branchial arches, lateral sclerotome, and limb buds of transgenic reporter mice at E9.5. From approximately E11.5, the expression of *Scx*-mScarlet was progressively confined to tendon tissue of the developing spine, tail, ribs, and extremities. (Figure 2A–C). At E15.5, the region of mScarlet expression became more localized to specific regions in the hind paw (Figure 2(D3)) and tail (Figure 2(D4)) with individual tendons. The expression of transgenes in individual tendons of the hind paw (Figure 2(D1)) and tail (Figure 2(D2)) became more specific at E18,5 (red signals were clearly detected in annulus fibrosus of the skinned tail). Lateral views of the *Scx*-mScarlet embryo at E15.5 indicate the developing tendons of limbs extends in the dorsal to ventral direction (Figure 2(E3)), and ligaments in the cranial region outline the ipsilateral masseter and temporalis muscles (Figure 2(E5)) [15]. Magnified images of the forelimb and hindlimb show the mScarlet-expressing tendons are widely involved in connecting of skeletal elements and muscles (Figure 2(E1,E2)). Magnified images of the back show the mScarlet-expressing connective tissue connecting the ribs to the intercostal muscles in the spinal area (Figure 2(E4)).

The expression analysis of *Scx*-mScarlet using the binocular microscope shows the ~13 kb genomic region from the mouse *Scx* gene in a bacterial artificial chromosome (BAC) recapitulate tendon atlas faithfully at most stages [16]. The fluorescence in *Scx*-mScarlet transgenic mouse expresses stably, which makes it suitable for monitoring Scx+ cells during tendon development and growth in vivo. It also makes the analysis of tendon tissue integrity easier as a suitable model for studying tendon injury.

#### 2.2.2. Expression of *Col1a1*-BFP and *Scx*-mScarlet Were Comparable

**Figure 3:**
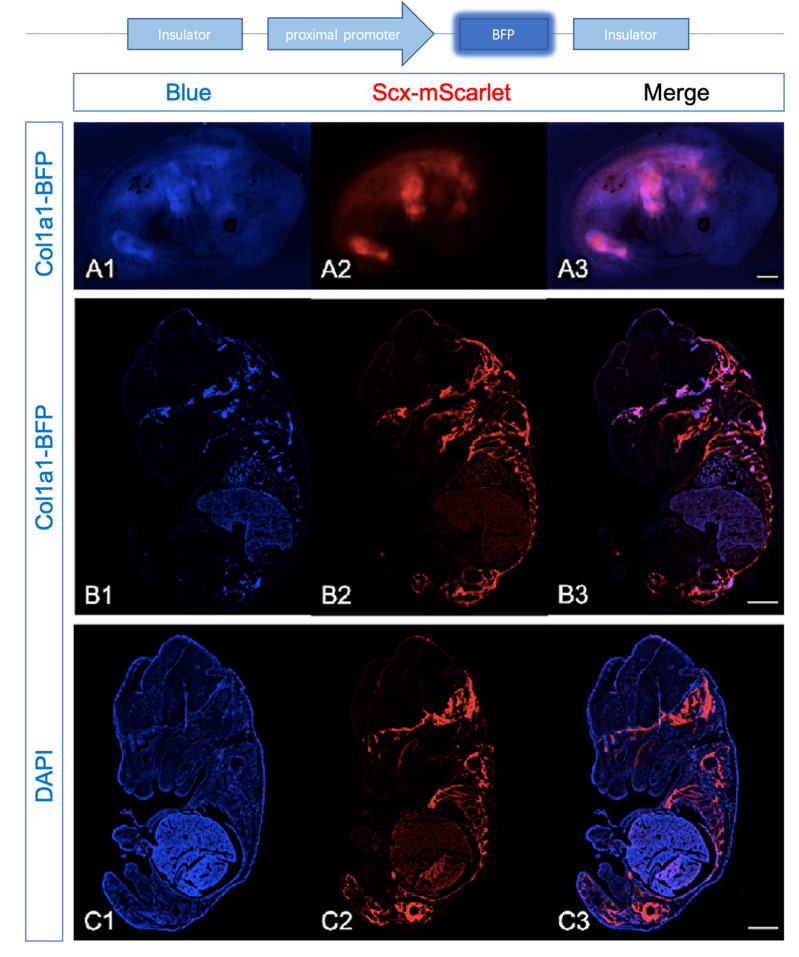
Areas of *Col1a1*-BFP expression coincided with *Scx*-mScarlet. To characterize transgenic mice, we detect the reporter signals of the whole-mount embryos using the binocular fluorescence microscope. Then, we also performed the analysis of reporters’ expressions in cryosections. The schematic drawing above illustrates the *Col1a1*-BFP transgene construct. (**A1–A3**): whole-mount images of a *Col1a1*-BFP; *Scx*-mScarlet embryos at E13.5. The expression of BFP (**A1**) and mScarlet (**A2**) were comparable. The merge (**A3**) of reporters’ expressions is mostly overlapping. (**B1**–**C3**): Frozen sagittal sections were prepared from *Col1a1*-BFP; *Scx*-mScarlet embryos at E15.5. *Col1a1*-BFP (**B1**) and *Scx*-mScarlet (**B2**,**C2**) in a frozen section of embryos at E15.5 were similar. (**B3**,**C3**): The merge of reporters’ expressions in a frozen section of embryos at E15.5 is mostly coinciding. (**C1**): DAPI (4′,6-diamidino-2-phenylindole) staining in a frozen section of embryos at E15.5. (Scale bar = 500 μm in (**A3**,**B3**,**C3**); arrows show connective tissues around the developing maxilla, basioccipital and basisphenoid bone; arrowhead indicates the connective tissues of mandible, tongue, and scapula).

Expression Profiles of *Scx*-mScarlet and *Col1a1*-BFP were largely overlapping in transgenic embryos at E13.5 (Figure 3(A1–A3)). Further analyses revealed that the signal of BFP under the fluorescence binocular microscope is weaker compared with mScarlet; we perfomed the analysis of reporters in cryosections.

The tendon-specific cis-acting elements (TSE1, TSE2) in pro-α1(I) gene can control the activity of the mouse *col1a1* promoter to induce the reporter specific in tendon fibroblasts [10,17]. To direct the reporters to be specifically expressed in the tendon, it was required to combine these cis-acting elements with osteoblasts specific elements [11,18]. Consequently, the reporters should express both in tendons and ossification centers [11]. In the craniofacial region of *Col1a1*-BFP, *Scx*-mScarlet embryos at E15.5, the expression of *Col1a1*-BFP was detected in the developing maxilla, basioccipital, and basisphenoid bone (Figure 3(B1)). The expression of *Scx*-mScarlet was more broadly detected than *col1a1*-BFP, reflecting the activity of *Scx*-BFP was weaker compared with mScarlet (Figure 3(B1–B3)). TagBFP shares similar bands of DAPI, so we stained DAPI in a frozen section of embryos at E15.5. After comparison, both of reporters expressed obviously in the regions of the mandible, tongue, scapula, ribs, and limbs (autofluorescence signals in the liver were possibly related to inadequate embryoperfusion).

The expression analysis of *Col1a1*-BFP shows the ~3 kb genomic region contains TSE1 and TSE2 recapitulate collagen I expression in tendons and ossification centers obviously. This implies *Col1a1*-BFP reporter is suited for studying the collagen I production of tenocytes during tendon development, growth, and repair in vivo. In turn, it could make the analysis of tendon extracellular matrix homeostasis indirectly possible.

### 2.3. The Density of Scx+ Cells Declined in Achilles Tendon of Aged Mouse

**Figure 4:**
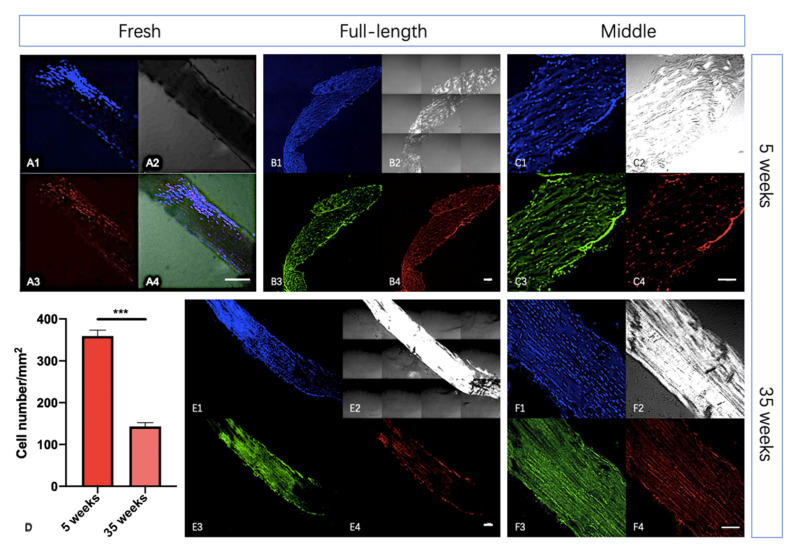
The Scx+ cell density in the central region of the Achilles tendon declined with mouse ageing. To further confirm the specificity of reporters’ expression, we analyzed the Achilles tendons in whole-mount and tissue sections. (**A1**–**A4**): *Col1a1*-BFP (**A1**), brightfield (**A2**), *Scx*-mScarlet (**A3**), and merge (**A4**) in a skinned Achilles tendon from a 5-week transgenic mouse. B–F: Longitudinal section of Achilles tendons stained with Scx antibody (**B3**,**C3**,**E3**,**F3**) and DAPI (**B1**,**C1**,**E1**,**F1**). Brightfield (**B2**,**C2**,**E2**,**F2**) and *Scx*-mScarlet (**B4**,**C4**,**E4**,**F4**) in the section show significant different. The length of Achilles tendons from 35-week mice (**E2**) was longer than the 5-week mice (**B2**). The Scx+ cell density in the central region of the Achilles tendon from 35-week mice (**F4**) was lower than the 5-week mice (**C4**). The lower-left schematic figure (**D**) indicates this difference is significant as determined by cell counting (Table S1; *** means *p* value ≤ 0.001). At least 50 lateral sections from 3 mice were quantified for each count. (Scale bar = 100 μm in (**A4**,**B4**,**C4**,**E4**,**F4**)).

To further confirm the specificity of *Col1a1*-BFP expression, we detected the blue signal of the fresh Achilles tendons from a 5-week transgenic mouse (Figure 4(A1–A4)). The *Col1a1*-BFP and *Scx*-mScarlet expressed robustly in tenocytes, reflecting faint BFP reporter expression in the embryos associated with different expression levels of *Col1a1* (Figure 3(B1–B3)). The *Scx*-mScarlet signal persists after birth and continues until at least 35-week-old (Figure 4E,F), but the intensity of mScarlet declines from 5 weeks (Figure 4C) to 35 weeks (Figure 4F). Scx antibody (Figure 4(B3,C3,E3,F3)) staining confirmed the *Scx*-mScarlet expression faithfully in tenocytes. The Scx+ cell density in the central region of the Achilles tendon from 35-week mice (Figure 4(F4)) was lower than that from the 5-week mice (Figure 4(C4)). To better quantify the cell density, we counted the cell number in the middle area of the full-length tendon section (Figure 4B,E). Because the Scx+ cells aggregated at the edge of the tendon section (cell boundaries are indistinguishable), we only counted the cells of tendon central region (Figure 4C,F; Figure S1; see Supplementary Materials). The results show that the density of Scx+ cells obviously decline in the Achilles tendon of the aged mice.

The expression analysis of *Col1a1*-BFP and *Scx*-mScarlet showed that the reporter signals are robustly expressed in tenocytes of the Achilles tendons from adult transgenic mice. This implies that our reporter mouse is might be well suited for studying cell re-differentiation and extracellular matrix alteration in vivo. The *Scx*-mScarlet is expressed until at least 35 weeks in mice. In addition, this makes the analysis of cell identity in aged tendons possible.

### 2.4. Tendon-Specific Double Reporter System Functioning Properly In Vitro

To better understand the expression patterns of tendon-specific double reporter system in vitro, we extracted adipose-derived mesenchymal stem cells (ADMSCs) from our transgenic mouse. After mechanical stimulation for 14 days, the expression of mScarlet and BFP increased.

**Figure 5:**
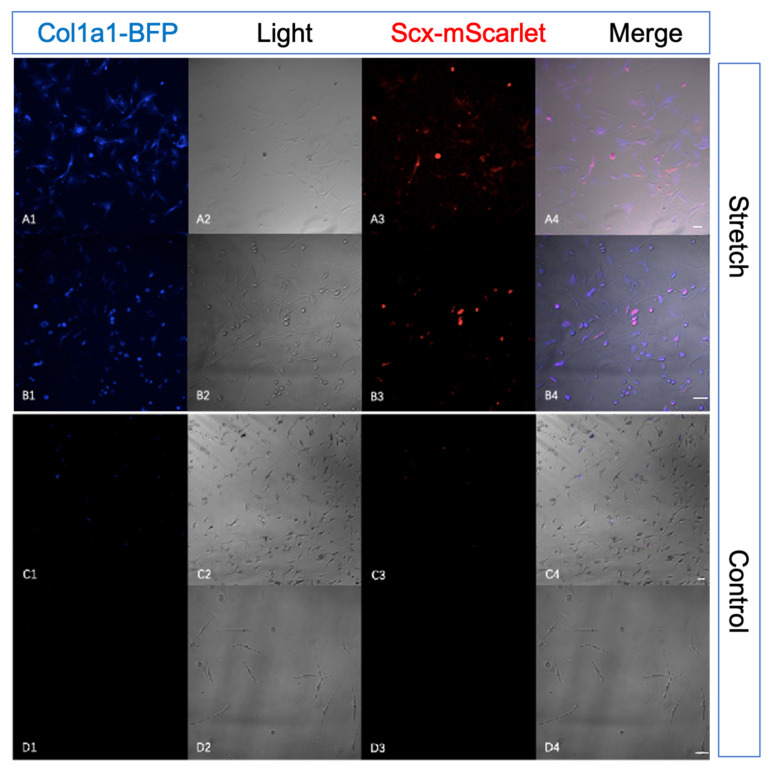
Mechanical stimulation increases the expression of mScarlet and BFP in adipose-derived mesenchymal stem cells (ADMSCs) in vitro. To validate the tendon-specific double reporter system by adipose-derived mesenchymal stem cells (ADMSCs) stretching assay in vitro, we harvested mesenchymal stem cells from adipose tissue of 10-week transgenic mice and transplanted them to silicone rubber membranes. After 0.5HZ mechanical stimulation 1 h per day for 14 days, the expression of mScarlet and BFP increased compared with cells cultured in normal 6-well plates under static conditions. (**A**,**B**): cells after mechanical stimulation (1 h per day, 5% stretch, 0.5 Hz), *Col1a1*-BFP (**A1**), brightfield (**A2**), *Scx*-mScarlet (**A3**), and merge (**A4**) were observed under a confocal microscope (×10 magnification). *Col1a1*-BFP (**B1**), brightfield (**B2**), *Scx*-mScarlet (**B3**), and merge (**B4**) were observed under a confocal microscope (×20 magnification). (**C**,**D**): culture the seeded membranes in normal 6-well plates as the control group, *Col1a1*-BFP (**C1**), brightfield (**C2**), *Scx*-mScarlet (**C3**), and merge (**C4**) were observed under a confocal microscope (×10 magnification). *Col1a1*-BFP (**D1**), brightfield (**D2**), *Scx*-Scarlett (**D3**), and merge (**D4**) were observed under a confocal microscope (×20 magnification). Experiments were repeated three times with cells from different donors independently. (Scale bar = 100 μm in (**A4**,**B4**,**C4**,**D4**)).

**Figure 6:**
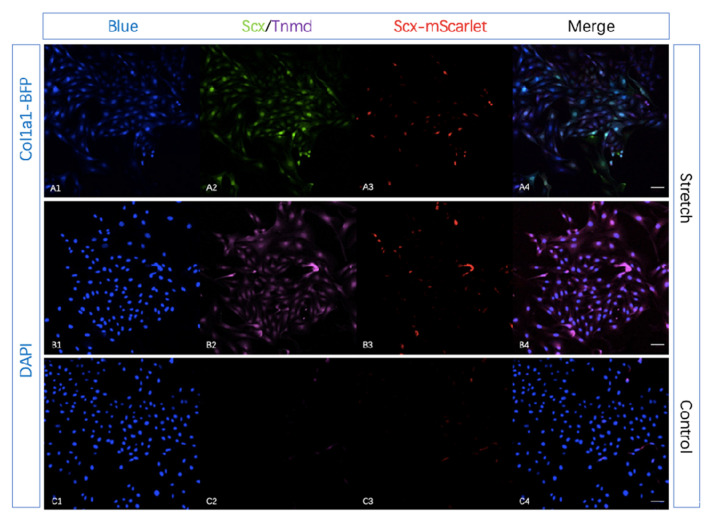
The Scx and Tnmd protein expression of cells seeded in the silicone rubber membranes increased. To confirm the increased expression of *Scx*-mScarlet and *Col1a1*-BFP in adipose-derived mesenchymal stem cells (ADMSCs) after mechanical stimulation, we performed immunohistochemical staining (IHC) using antibodies against Scx and Tnmd. (**A**,**B**): cells after mechanical stimulation (1 h per day, 5% stretch, 0.5 Hz), *Col1a1*-BFP (**A1**), immunofluorescent staining for Scx (**A2**), *Scx*-mScarlet (**A3**), and merge (**A4**) were observed under a confocal microscope (×20 magnification). DAPI staining (**B1**), immunofluorescent staining for Scx (**B2**), *Scx*-mScarlet (**B3**), and merge (**B4**) were observed under a confocal microscope (×20 magnification). (**C**): culture the seeded membranes in normal 6-well plates as the control group, DAPI staining (**C1**), immunofluorescent staining for Tnmd (**C2**), *Scx*-mScarlet (**C3**), and merge (**C4**) were observed under a confocal microscope (×20 magnification). Experiments were repeated three times with cells from different donors independently. (Scale bar = 50 μm in (**A4**,**B4**,**C4**,**D4**)).

To validate the tendon-specific double reporter system by adipose-derived mesenchymal stem cells (ADMSCs) stretching assay in vitro, we harvested mesenchymal stem cells from adipose tissue of 10-week transgenic mice and seeded them on silicone rubber membranes with type I Collagen coating. After 0.5 HZ mechanical stimulation (5% stretch) 1 h per day for 14 days (medium contain 10 ng/mL TGFβ3), the expression of mScarlet and BFP increased compared with seeded cells culture in normal 6-well plates (Figure 5, Figure 6(A1,A3)). To confirm the increased expression of *Scx*-mScarlet and *Col1a1*-BFP in adipose-derived mesenchymal stem cells (ADMSCs) after mechanical stimulation, we performed the immunohistochemical staining (IHC) using antibodies against Scx and Tnmd. Compared with the control group, the stretching group clearly showed positive Scx and Tnmd staining (Figure 6(A2,B2)). This revealed that mechanical force in combination with medium contain TGF-β3 (ransforming growth factor beta-3) clearly up-regulated the expression of Scx and Tnmd.

We validated the tendon-specific double reporter system via an in vitro stretching experiment. The expression analysis of *Col1a1*-BFP, *Scx*-mScarlet, Scx and Tnmd show that the reporters’ signals faithfully recapitulate the activity of *Scx* and Tnmd in vitro. This implies that our reporter mouse lines are suited for studying tendon-specific cell re-differentiation and extracellular matrix alteration in vitro.

## 3. Discussion

We generated and characterized a novel tendon-specific double reporter transgenic mouse line, which allows the study of cell differentiation/re-differentiation and extracellular matrix alteration of tendons in vitro and in vivo.

As the density of the cells in the tendon decreases postnatally, even the signal of *Scx*GFP became undetectable by 8 months [8]. So, we analyzed the signals of *Col1a1*-BFP and *Scx*-mScarlet in Achilles tendons from a 35-week transgenic mouse. The results demonstrated that *Col1a1*-BFP and *Scx*-mScarlet were detectable in 35-week mice, although the autofluorescence became stronger. This shows that the transgenic mouse line has the potential to be used for studies of tendon aging.

It will be interesting to detect the *Col1a1*-BFP and *Scx*-mScarlet in the tendon from much older mice in the future. To quantify the change of cell density during ageing, we counted the cell number in the central region of the Achilles tendon. Because the Scx+ cells aggregated at the edge of the tendon section, we utilized the count method from Toru et al. [19]. This result provides an initial baseline for tendon ageing study.

Tendon stem/progenitor cells (TSPCs) are a particular cell population with the ability to repair tendons [20]; Harvey et al. first reported that Tppp3+Pdgfra+ fibro-adipogenic progenitors as tendon stem cells are located in the surrounding tissues of tendons(sheath) [21]. Additionally, adipose tissues are an ideal source of cells for the treatment of tendon injuries [22]. With mechanical stimulation [23,24], growth factors [22,23], and hypoxia [25], adipose-derived mesenchymal stem cells (ADMSCs) show a potential for tendon healing in vitro and in vivo [26]. We harvested ADMSCs from adipose tissue adjacent to the tendons like the infrapatellar fat pad and the Kagers fat pad [27]. Then we seeded the cells to silicone rubber membranes with type I Collagen coating for stretching. The results of IHC and reporters showed that a combination of mechanical stimulation, hypoxia, and TGF-β3 clearly up-regulated the expression of tendon specific markers like Scx and Tnmd in these cells.

Furthermore, our results suggest that our tendon-specific double reporter transgenic mouse is a novel model which could be used to monitor tenocytes’ lineage and functions alteration in vitro and in vivo.

## 4. Materials and Methods

### 4.1. Cloning of the Scx-mScarlet-2A-rtTAV16 and Col1a1-BFP Transgenic Constructs

The *Col1a1* proximal promoter DNA from BAC CLONE *Col1a1* (BACPAC Genomics, Emeryville, CA, USA) was amplified in Q5 High-Fidelity 2X Master Mix (NEB #M0492, New England Biolabs, MA, USA), then inserted in a vector with Insulator by NEBuilder HiFi DNA Assembly Master Mix (NEB #E2621, New England Biolabs, MA, USA). Using the same strategy, TagBFP fragment was inserted to obtain a recombinant plasmid containing Insulators, proximal promoter, and TagBFP. The locus of TagBFP is in the downstream of the ATG site (the start codon encodes the amino acid).

The *Scx* promoter DNA in BAC CLONE *SCX* (BACPAC Genomics, Emeryville, CA, USA) was amplified in Q5 High Fidelity 2X Master Mix, then inserted in a vector with mScarlet by NEBuilder HiFi DNA Assembly Master Mix. Using the same strategy, the downstream fragment of the first exon of *Scx* was inserted. The locus of mScarlet is between the two exons of the *Scx* in the transgenic construct.

After being linearized with EcoRV (R0195S, New England Biolabs, MA, USA) + SalI (R0138S, New England Biolabs, MA, USA) and AscI (R0558S, New England Biolabs, MA, USA) + FseI (R0588S, New England Biolabs, MA, USA) separately, the *col1a1*-BFP and *Scx*-mScarlet transgenic constructs were isolated for prenuclear micro-injection.

### 4.2. Mechanical Stimulation In Vivo

Adipose-derived mesenchymal stem cells (ADMSCs) were isolated according to the protocol from Seluanov et al. [28]; the adipose tissue around the tendons, like the infrapatellar fat pad and the Kagers fat pad, were harvested from transgenic mice. The tissue fragments were transferred into 2 mL of medium (DMEM, supplemented with 20% FBS, penicillin/streptomycin, and Amphotericin B). Then, we added 200 µL of Liberase Blendzyme III (Roch, Basel, Schweiz) stock solution (10 mg/mL) to the medium and incubated it overnight in a cell incubator. We centrifuged the cells and tissue debris at 1200 rpm for 5 min to precipitate the cells and tissue debris. The supernatant was discarded and the mixture, including the tissue debris, was transferred into 2 mL of medium (DMEM, supplemented with 20% FBS, penicillin/streptomycin, Amphotericin B and Doxycycline). We replaced the medium with fresh medium every day until all tissue debris had been washed away. Then, we seeded the cells to silicone rubber membranes with type I Collagen coating for stretching (1 h per day, 5% stretch, 0.5 Hz). The seeded membranes were cultured in normal 6-well plates as the control group, and another group of seeded membranes in a stretching chamber as the stretch group (Videos S1 ans S2; medium contain 10 ng/mL TGFβ3, Peprotech, #100-36E, Rocky Hill, NJ, USA). The chamber was sealed with parafilm to create a hypoxic environment similar in vivo.

### 4.3. Immunohistochemistry and Immunocytochemical Staining

The 5–35-weeks old mice were anesthetized with isoflurane and executed by cervical dissection. The carcasses were disinfected with alcohol, and the skin of the Achilles tendon area of the mice was incised to expose the Achilles tendon. The Achilles tendon was cut after it was peeled out. We set the mice mating and checked the time of emergence of the plug to calculate the age of the embryo. Pregnant mice were anesthetized with isoflurane and sacrificed by cervical dissection. Embryos of E10.5, E12.5, and E14.5 in utero were detached on ice, placed in pre-chilled PBS, and the fetal membranes were reserved for genotyping. All operations were performed after completing a training course and obtaining permission, and all steps complied with the requirements of the Animal Welfare Act (Tierschutzgesetz). Samples washed in PBS were fixed overnight at 4 °C in freshly prepared 4% PFA solution. We washed the samples well three times in PBS at 4 °C for 1 h each. Whole-mount embryos were photographed by a binocular microscope. Immersion in 10%, 20%, and 30% sucrose solutions was done for pre-embedding protection of cryosections. Then, we embedded them in OCT and placed on dry ice to solidify long-term storage in a −80 °C refrigerator. The tissue blocks were left overnight in the −20 °C refrigerator for sectioning the next day. We adjusted the temperature of the Cryostat microtome (Leica CM3050S) and trimmed the tissue block. Continuous sectioning of embryos at 10 µm thickness. We mounted the cut tissue pieces on SuperFrost slides and performed Confocal Microscope (Carl Zeiss LSM900) for photography and stored them at −20 °C away from light.

The slides were washed three times with PBS to remove impurities. We fixed the cells with 4% PFA for 30 min at room temperature. Then, the cells were washed three times with PBS for 10 min each. The cells were permeabilized in PBS containing 0.1% Triton X-100 for 15 min to permeabilize the cell membrane. Block endogenous cellular antigens with 5% BSA for 1 h at room temperature. Cells were submerged in diluted primary antibodies (Scx: ab58655 and Tnmd: ab203676, Abcam, Berlin, Germany) and incubated overnight at 4 °C. The cells were washed three times with PBS for 10 min each. Cells were submerged in diluted secondary antibodies and incubated for 1 h at room temperature. Then, cells were washed three times with PBS for 10 min each time. DAPI was added and stained for 10 min for nuclear staining. Cells were washed three times with PBS for 10 min each. After flow-through rinsing, Coverslip slides with Mowiol 4–88 (Roche, #0718, Basel, Schweiz) were used for examination.

## Data Availability

The datasets generated and analyzed during the current study are available from the corresponding authors on reasonable request.

## Supplementary Materials

The following are available online at https://www.mdpi.com/article/10.3390/ijms222011189/s1, Figure S1: Cell counting area, Table S1: Cell numbers, Videos S1 and S2: Stretching system.
