# Supplementary material for: A Tendon-Specific Double Reporter Transgenic Mouse Enables Tracking Cell Lineage and Functions Alteration In Vitro and In Vivo
Source: Int J Mol Sci. 2021 Oct 17;22(20):11189. doi: 10.3390/ijms222011189 (PMC8537162; doi:10.3390/ijms222011189)
Supplement: Supplementary file 1 [file ijms-22-11189-s001.zip › Figure S1 Cell counting area.pdf]

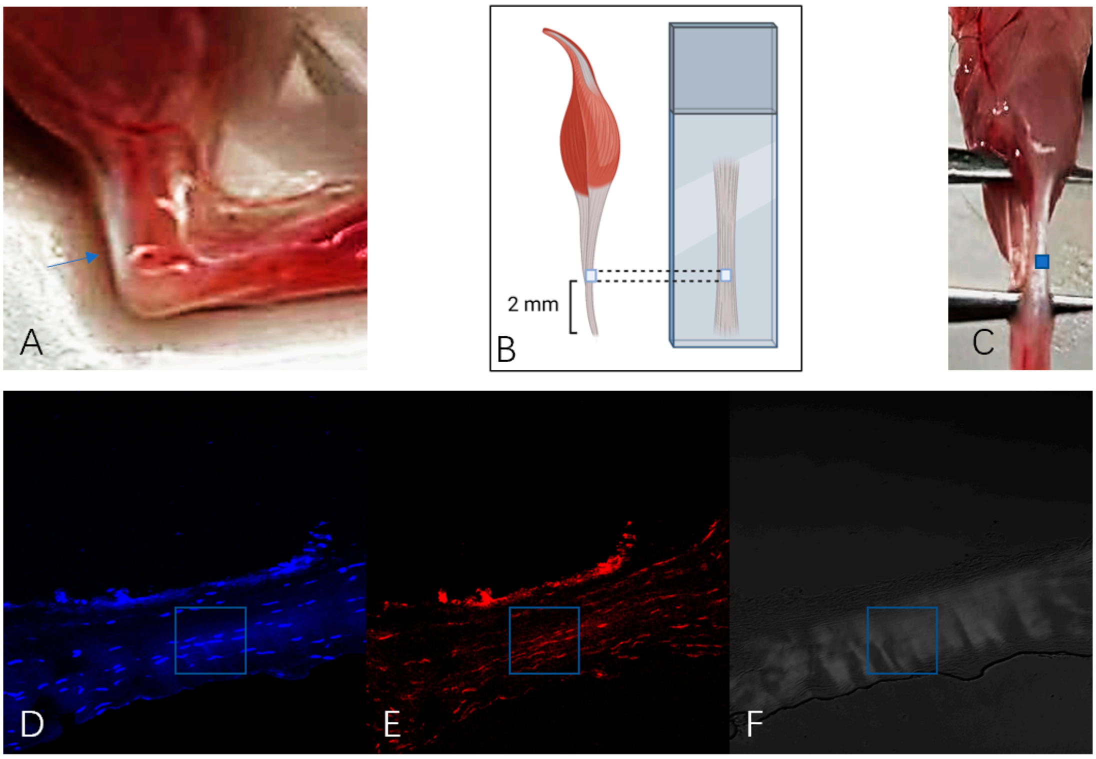


**Figure S1.** The cell counting area is based on the counting method from Toru et al. [19]. The arrow shows the Achilles tendon (A). Then we harvested the tendon and prepared the sections (B). The cell counting area is located 2 mm proximal to the calcaneus (C). Because the Scx+ cells (blue, DAPI; red, ScxScarlet; grey, brightfield) aggregated at the edge of the tendon section (cell boundaries are indistinguishable), we only counted the cells of the central tendon region (squares in figure D, E, F).
